# Interactive effects of diabetes and impaired kidney function on cognitive performance in old age: a population-based study

**DOI:** 10.1186/s12877-016-0193-6

**Published:** 2016-01-12

**Authors:** Zhaoxue Yin, Zhongrui Yan, Yajun Liang, Hui Jiang, Chuanzhu Cai, Aiqin Song, Lei Feng, Chengxuan Qiu

**Affiliations:** Division of Non-communicable Diseases Control and Community Health, Chinese Center for Disease Control and Prevention, Beijing, China; Department of Neurology, Jining No. 1 People’s Hospital, Shandong, China; School of Public Health, Jining Medical University, Shandong, China; Aging Research Center, Department of Neurobiology, Care Sciences and Society, Karolinska Institutet-Stockholm University, Stockholm, Sweden; Xing Long Zhuang Hospital, Yankuang Group, Shandong, China; Department of Psychological Medicine, National University of Singapore, NUHS Tower Block, 1E Kent Ridge Road, Singapore, Singapore

**Keywords:** Diabetes, Kidney function, Cognitive impairment, Aging, Population-based study

## Abstract

**Background:**

The interactive effect between diabetes and impaired kidney function on cognitive impairment in older adults has not yet been reported. The aim of this study was to investigate the association of diabetes and impaired kidney function with cognitive impairment among Chinese older people living in a rural area.

**Methods:**

This cross-sectional study included 1,358 participants (age ≥60 years; 60.5 % women) in the population-based Confucius Hometown Aging Project in Shandong, China. Data on demographics, lifestyle factors, health history, use of medications, global cognitive function, and kidney function were collected through structured interviews, clinical examinations, and blood tests. We defined diabetes as a fasting plasma glucose level ≥7.0 mmol/l or use of hypoglycemic agents, impaired kidney function as glomerular filtration rate estimated from cystatin C (eGFR_cys_) <60 ml/min/1.73 m^2^. Cognitive impairment was defined using the education-based cut-off scores of Mini-Mental State Examination (MMSE). Data were analyzed using multiple general linear and logistic regression models.

**Results:**

Cognitive impairment was defined in 197 (14.5 %) persons. The multi-adjusted β coefficient of MMSE score associated with diabetes was −0.06 (95 % confidence interval [CI], −0.16, 0.03); the corresponding figures associated with eGFR_cys_ <60, 60–89.9, and ≥90 ml/min/1.73 m^2^ were −0.15 (−0.28, −0.02), −0.01 (−0.10, 0.08), and 0 (reference) (*P*_trend_ = 0.046), respectively. Diabetes and impaired kidney function showed an interactive effect on cognitive impairment (*P*_interaction_ = 0.02). Compared with individuals having neither diabetes nor impaired kidney function, those with both conditions had a multi-adjusted odds ratio of 4.23 (95 % CI, 2.10–8.49) for cognitive impairment. The relative excess risk due to interaction was 2.74.

**Conclusions:**

This study suggests that concurrent presence of diabetes and impaired kidney function is associated with a substantial likelihood for cognitive impairment in older adults.

## Background

In 2010, ~36 million people were living with dementia in the world; the number was projected to nearly double every 20 years [1]. In China, the age-specific prevalence of dementia had steadily increased during 1990–2010, and ~9.2 million people were affected by dementia in 2010 [2]. Furthermore, mild cognitive impairment affects ~21 % of Chinese people aged 65 years or older, with the prevalence being higher in rural than in urban residents [3]. Dementia or cognitive impairment is the major cause of functional dependence and institutionalization, which significantly decreases quality of life and increases costs of social care. Identifying modifiable risk factors for cognitive impairment or dementia would foster the development of proper strategies for intervention, thus reducing the individual and societal burden of the most devastating mental disorders in the aging society.

Diabetes affects about one in five Chinese people aged 60 years or older [4, 5]. In the past decade, numerous population-based studies have shown rather consistent association of diabetes with cognitive impairment and dementia [6, 7]. In addition, chronic kidney disease or reduced kidney function is common among older people [8]. Cystatin C, a marker of kidney function, is considered superior to serum creatine in the general population of older people [9]. Evidence has emerged that reduced kidney function, measured with markers of low glomerular filtration rate, is associated with cognitive impairment in older adults [10–12]. As diabetes is one of the leading causes of chronic kidney disease, older adults with both diabetes and reduced kidney function may present substantial impairment in cognitive performance. However, the potential interaction between diabetes and impaired kidney function on cognitive impairment in older adults has not yet been reported.

On the basis of previous research, we hypothesize that diabetes and reduced kidney function can interact to affect cognitive performance among older people, such that older adults with both diabetes and impaired kidney function would have substantially increased likelihood of cognitive impairment compare to those with neither condition. In this study, we seek to test this hypothesis using data from a population-based study of Chinese older people living in a rural community.

## Methods

### Study population

Participants were from the Confucius Hometown Aging Project (CHAP). The aim of CHAP was to investigate the role of vascular risk factors and atherosclerotic mechanisms in aging and health among older people (age ≥60 years) living in the Xing Long Zhuang community nearby Qufu city (Hometown of Confucius) in Shandong, China, as fully described elsewhere [5, 13]. In brief, of the 1,743 eligible subjects who were invited to participate in the survey in 2010–2011, 205 refused, lost contact or died before the survey, and 180 had missing data on fasting plasma glucose (FPG) (*n* = 17) or cystatin C (*n* = 163). Thus, the analytical sample included 1,358 persons (77.9 % of all eligible subjects).

The CHAP protocols were reviewed and approved by the ethics committee of Jining No. 1 People’s Hospital at Jining Medical University, Shandong. Written informed consent was obtained from all participants or, in the case of cognitively impaired persons, from the next of kin.

### Data collection and definition of measurements

Data were collected at the local Xing Long Zhuang Hospital by trained physicians and nurses, following a standard questionnaire that was developed from the WHO STEPwise approach to Surveillance and the Study on Global Ageing and Adult Health [5, 13, 14]. We collected data on demographics, lifestyles (e.g., smoking, alcohol consumption, and physical activity), medical history (e.g., hypertension, diabetes, and stroke), and use of medications (e.g., antihypertensive drugs and hypoglycemic agents). Weight and height were measured with subjects wearing light clothes with no shoes. Arterial blood pressure was measured twice on the right arm using a sphygmomanometer in the sitting position after at least a five-min rest, and the mean of the two readings was used for analysis. We defined and categorized various covariates as previously reported [5, 13]. Smoking status was defined as “Yes” if the subjects consumed cigarette every day or almost every day currently, otherwise the smoking status was defined as “No”. Alcohol drinking was defined as “No” if the subjects did not drink alcohol or drank less than one time per month, and “Yes” if the subjects drank alcohol one time per month or more in the last year. Physical exercise was referred to as regularly engaging moderate or heavy intensity exercise at least 3 times a week, and lasting for 20 min and over each time. Hypertension was defined as blood pressure ≥140/90 mmHg or current use of antihypertensive drugs. Body mass index (BMI) was calculated as weight (kilograms) divided by height (meters) squared. The 15-item Geriatric Depression Scale (GDS-15) was used to assess depressive symptoms, and the presence of high depressive symptoms was defined as a GDS-15 score ≥5 [15].

Peripheral blood samples were taken after an overnight fast. Total cholesterol, FPG, and serum cystatin C were measured using the enzymatic methods by an Automatic Biochemistry Analyzer (Olympus AU400, Japan) in the certified hospital laboratory.

Diabetes was defined as FPG ≥7.0 mmol/l or current use of hypoglycemic agents or insulin injection. Kidney function was assessed by the cystatin C-based estimated glomerular filtration rate (eGFR_cys_), which was calculated following the equation proposed by the Chronic Kidney Disease Epidemiology Collaboration [16]: eGFR_cys_ = 133 × (cystatin C/0.8)^−1.328^ × 0.996^age^ (×0.932 if female) for cystatin C ≥0.8 mg/l; 133 × (cystatin C/0.8)^−0.499^ × 0.996^age^ (×0.932 if female) for cystatin C <0.8 mg/l. We first classified eGFR_cys_ into <60, 60–89.9, and ≥90 ml/min/1.73 m^2^, and then we defined impaired or reduced kidney function as eGFR_cys_ <60 ml/min/1.73 m^2^ [17].

Global cognitive functioning was assessed using a Chinese version of the Mini-Mental State Examination (MMSE) that had been validated among Chinese elderly population [18]. Cognitive impairment was defined using education-based cut-off points of MMSE [19]: 19/20 for persons without formal education (illiteracy), 22/23 for those with 1–6 years of education (primary school), and 26/27 for those with more than 6 years of education (middle school and higher).

### Statistical analysis

Characteristics of study participants by cognitive impairment were compared using *t*-test for continuous variable and chi-square test for categorized variables. We used general linear regression analysis to estimate the β coefficient and 95 % confidence interval (CI) of MMSE score associated with diabetes and impaired kidney function, in which the MMSE score was transformed to –log (31-MMSE score) owing to negatively skewed distribution of the original MMSE score [20]. We employed logistic regression models to estimate the odds ratio (OR) and 95 % CI of cognitive impairment associated with diabetes and impaired kidney function. We further assessed statistical interaction between diabetes and kidney function on cognitive performance by simultaneously including the two independent variables and their cross-product term in a same model. When a potential statistical interaction was detected, stratified analysis was further conducted to assess the joint effect of diabetes and impaired kidney function on cognitive performance, in which we categorized the subjects into four groups, that is, having neither diabetes nor impaired kidney function (reference), having diabetes alone, having impaired kidney function alone, and having both conditions. Relative excess risk due to interaction that reflects the departure from an additive interactive effect on a relative risk scale was calculated using the formula [21]: OR_AB_–OR_A_–OR_B_ + 1. We reported the main results from two models: model 1 was adjusted for age, sex, and years of education, and model 2 was further adjusted for smoking, alcohol drinking, physical activity, BMI, hypertension, total cholesterol, stroke, and depressive symptoms.

SAS 9.1.3 (SAS Institute Inc., Cary, NC, USA) was used for all statistical analyses. We considered *P* < 0.05 to be statistically significant in a two-sided test.

## Results

### Characteristics of study participants

The mean age of the 1,358 participants was 68.6 years (SD, 5.0) and 60.5 % were women. Compared with subjects without cognitive impairment, those with cognitive impairment were older, more likely to be female, illiterate, and physically inactive, less likely to drink alcohol, and had a higher level of serum total cholesterol, FPG, and a lower level of eGFR_cys_, as well as higher prevalence of self-reported stroke, depressive symptoms, and diabetes (Table 1).

**Table 1:** Characteristics of study participants by cognitive impairment

		Cognitive impairment		
Characteristics	Total sample	No	Yes	*P*-value
No. of subjects	1,358	1,161	197	
Age (years), mean (SD)	68.6 (5.0)	68.23 (4.8)	70.90 (5.44)	<0.001
Female, n (%)	821 (60.5)	671 (57.80)	150 (76.14)	<0.001
Education level, n (%)				
Illiterate	370 (27.25)	294 (25.32)	76 (38.58)	
1–6 years	763 (56.19)	657 (56.59)	106 (53.81)	
≥7 years	225 (16.57)	210 (18.09)	15 (7.61)	<0.001
Current smoking, n (%)	185 (13.62)	164 (14.13)	21 (10.66)	0.190
Alcohol drinking, n (%)	276 (20.63)	250 (21.87)	26 (13.33)	0.007
Physical activity, n (%)	214 (15.76)	195 (16. 80)	19 (9.64)	0.01
Stroke, n (%)	86 (6.33)	59 (5.08)	27 (13.71)	<0.001
Hypertension, n (%)	1,072 (78.94)	914 (78.73)	158 (80.20)	0.640
Total cholesterol (mmol/l), mean (SD)	5.33 (1.05)	5.29 (1.04)	5.53 (1.09)	0.004
BMI (kg/m^2^), mean (SD)	26.29 (3.76)	26.30 (3.68)	26.27 (4.20)	0.930
Depressive symptom, n (%)	271 (19.96)	213 (18.35)	58 (29.44)	<0.001
Diabetes, n (%)	283 (20.84)	226 (19.47)	57 (28.93)	0.003
FPG (mmol/l), mean (SD)	5.70 (1.63)	5.66 (1.58)	6.02 (1.87)	0.004
eGFR_cys_ (ml/min/1.73 m^2^), mean (SD)	78.30 (20.06)	79.38 (20.02)	71.95 (19.18)	<0.001

*SD* standard deviation, *FPG* fasting plasma glucose, *BMI* body mass index, *eGFR*_*cys*_ cystatin C-based estimated glomerular filtration rate

### Independent effect of diabetes and impaired kidney function on cognitive impairment

When the transformed MMSE score was analyzed as a continuous variable (outcome), diabetes was significantly associated with a lower transformed MMSE score in model 1, but the association was not significant when additional variables were included in model 2 (Table 2). There was a linear trend towards a decreasing level of eGFR_cys_ being associated with a decreasing transformed MMSE score (multi-adjusted *P*_trend_ = 0.046); the presence of impaired kidney function (eGFR_cys_ <60 ml/min/1.73 m^2^) was significantly associated with a lower transformed MMSE score (Table 2).

**Table 2:** Association of diabetes and eGFR_cys_ with the Mini-Mental State Examination (MMSE) score and cognitive impairment

	β coefficient (95 % CI) of MMSE score^a^		Odds ratio (95 % CI) of cognitive impairment	
Factors	Model 1^b^	Model 2^b^	Model 1^b^	Model 2^b^
Diabetes	−0.09 (−0.18,−0.0004)	−0.06 (−0.16, 0.03)	1.81 (1.27, 2.60)	1.75 (1.20, 2.53)
eGFR_cys_, ml/min/1.73 m^2^				
≥90	0 (reference)	0 (reference)	1.00 (reference)	1.00 (reference)
60–89.9	−0.004 (−0.10, 0.09)	−0.01 (−0.10, 0.08)	0.99 (0.64, 1.52)	0.98 (0.63, 1.52)
<60	−0.15 (−0.28, −0.02)	−0.15 (−0.28, −0.02)	1.32 (0.78, 2.22)	1.37 (0.80, 2.35)
*p*_trend_	0.039	0.046	0.27	0.23

*CI* confidence interval, *eGFR*_*cys*_ cystatin C-based estimated glomerular filtration rate, *MMSE* Mini-Mental State Examination

^a^The original MMSE score was transformed as –log (31-MMSE score)

^b^Model 1 was adjusted for age, sex, and years of education, and model 2 was further adjusted for smoking, physical activity, alcohol drinking, body mass index, total cholesterol, hypertension, stroke, and depressive symptoms

When global cognitive functioning was analyzed as a dichotomous variable (i.e., cognitive impairment, yes vs. no) in the logistic model, diabetes was significantly associated with an increased likelihood of cognitive impairment, even in model 2 when all the examined potential confounders were taken into account. However, there was no significant linear trend for the association between levels of eGFR_cys_ and likelihood of cognitive impairment, although eGFR_cys_ <60 ml/minute/1.73 m^2^ tended to be associated with an elevated likelihood of cognitive impairment (Table 2). When kidney function was dichotomized into impaired vs. non-impaired (i.e., eGFR_cys_ <60 vs. ≥60 ml/minute/1.73 m^2^), the multi-adjusted β coefficient (95 % CI) of the transformed MMSE score associated with impaired kidney function was −0.15 (−0.25, −0.04) (*P* = 0.007) in model 1 and -0.14 (−0.25, −0.03) (*P* = 0.01) in model 2, whereas the OR (95 % CI) of cognitive impairment associated with impaired kidney function was 1.33 (0.90–1.96) (*P* = 0.15) in model 1 and 1.40 (0.93–2.09) (*P* = 0.11) in model 2.

### Joint impacts of diabetes and impaired kidney function on cognitive impairment

**Fig. 1:**
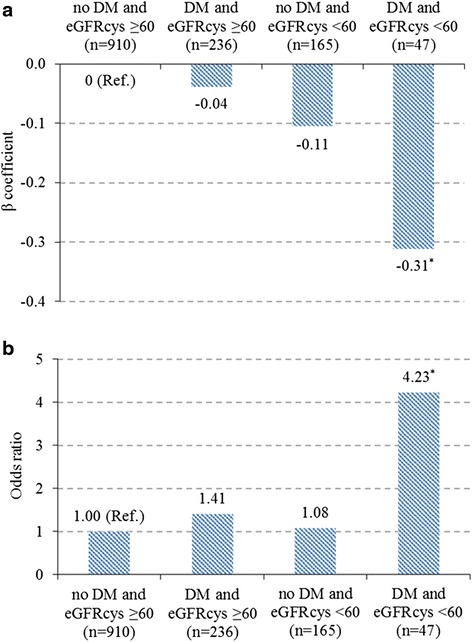
Joint impacts of diabetes and impaired kidney function on the transformed MMSE score (**a**) and global cognitive impairment (**b**). β coefficients were derived from general linear regression model (**a**), and odds ratios from logistic regression model (**b**) while controlling for age, sex, years of education, smoking, physical activity, alcohol drinking, body mass index, total cholesterol, hypertension, stroke, and depressive symptoms. DM, diabetes mellitus; eGFR_cys_, cystatin C-based estimated glomerular filtration rate; MMSE, Mini-Mental State Examination. ^*^ *P* < 0.01

When the transformed MMSE score was analyzed as a continuous variable, there was no statistically significant interaction between diabetes and impaired kidney function on global cognitive performance (*P*_interaction_ = 0.19). Stratified analysis by diabetes and impaired kidney function showed that the multi-adjusted β coefficient (95 % CI) of the transformed MMSE score associated with having neither diabetes nor impaired kidney function, having only diabetes, having only impaired kidney function, and having both diabetes and impaired kidney function was 0 (reference), −0.04 (−0.14, 0.06), −0.11 (−0.23, 0.02), and −0.31 (−0.52, −0.10), respectively (Fig. 1a). However, when cognitive function was examined as a dichotomous variable (i.e., cognitive impairment, yes vs. no), there was a statistically significant interaction of diabetes with impaired kidney function (eGFR_cys_ <60 ml/minute/1.73 m^2^) on cognitive impairment (*P*_interaction_ = 0.02). Further analysis stratified by diabetes and impaired kidney function suggested that in comparison with persons having neither diabetes nor impaired kidney function, the OR of having cognitive impairment for those with both conditions was 4.23 (95 % CI, 2.10–8.49) (Fig. 1b); the relative excess risk due to interaction was 2.74.

Similar results were obtained when subjects with a history of stroke (*n* = 86) were excluded from the analytical sample (data not shown).

## Discussion

This population-based study of Chinese elderly people living in a rural community suggested that diabetes and impaired kidney function were associated with poor performance in global cognitive test or an increased likelihood of cognitive impairment. In particular, this study revealed an interactive effect between diabetes and impaired kidney function on global cognitive impairment, such that older individuals with both diabetes and impaired kidney function showed a substantially increased likelihood of cognitive impairment.

The main effects of diabetes on cognitive impairment or dementia in old adults are consistent with numerous previous studies [6, 22–24]. Individuals with impaired kidney function did perform worse in global cognitive test compared to persons with normal kidney function, which was in line with earlier studies [10, 11]. However, when cognitive performance was dichotomized into cognitive impairment vs. non-impairment, the association between impaired kidney function and cognitive impairment appeared to become less evident. This is likely due to the fact that categorizing a continuous outcome may reduce statistical power [25]. The key finding of our study refers to the detection of an interactive effect between diabetes and impaired kidney function on cognitive impairment, which may have implications for understanding the pathophysiology of cognitive consequence related to diabetes. Our study indicates that the association of diabetes with cognitive impairment is to some extent dependent on the presence of impairment in kidney function, such that diabetes was associated with cognitive impairment mainly in the presence of impaired kidney function. This implies that impaired or reduced kidney function may be a critical pathophysiological basis for older people with diabetes to manifest cognitive impairment or dementia. Chronic kidney disease is one of the most common complications of long-term diabetes. This is in line with previous studies showing that long-term or severe diabetes is important in the development of cognitive impairment and dementia [26, 27].

Glucose toxicity, inflammation, oxidative stress, insulin resistance, blood–brain barrier disruption, and cerebral macro- and microvascular disease associated with diabetes are the well-established pathophysiological mechanisms linking diabetes with cognitive impairment [7, 28]. Small vessel diseases in the brain and kidney share common causes or cardiometabolic risk factors such as diabetes and hypertension. Thus, impaired kidney function due to glomerular small vessel disease has been suggested to correlate with subclinical cerebral microvascular disease in older adults [29, 30], which, together with neuronal injury owing to uremic toxins, could promote the development of cognitive impairment and dementia [31]. The biological mechanisms for the interactive effect between diabetes and impaired kidney function on cognitive impairment are not fully understood, but multiple pathways are likely to be involved. First, diabetes and impaired kidney function potentially share common mechanisms leading to cognitive impairment such as inflammation, peripheral vascular disease, and cardiovascular disease [32]. Furthermore, the concurrent presence of diabetes and impaired kidney function may indicate substantial burden of cerebral small vessel disease, which might considerably increase likelihood of having cognitive impairment [29]. Finally, chronic kidney disease may accelerate cognitive decline in patients with diabetes, through pathophysiological processes such as anemia, inflammation or oxidative stress [33, 34].

A major strength of this study refers to the population-based design that targets older people living in a rural area in China to whom very little attention has been paid by researchers so far. Furthermore, we were able to control for a broad range of health-related factors and health conditions, as potential confounders, that were assessed from epidemiological, clinical, and biological aspects by local hospital staff. This study also has limitations. First, the cross-sectional study does not allow us to determine the temporal relationship of diabetes and impaired kidney function to cognitive impairment. Further, selective survival might affect the cross-sectional association, especially when diabetes, impaired kidney function, and cognitive impairment are differentially associated with survival. Third, we did not have a direct measure of glomerular filtration rate, although the cystatin C-based eGFR_cys_, as an approximation of glomerular filtration rate, is considered superior to that of the estimation from serum creatinine [9]. Finally, we did not examine the interactive effects of diabetes and impaired kidney function on specific domains of cognitive function due to lack of the relevant data.

## Conclusions

In conclusion, this cross-sectional study reveals a strong interactive effect between diabetes and impaired kidney function on global cognitive impairment among older adults. This implies that assessment of cognitive function should be routinely recommended within geriatric settings for elderly patients with diabetes who also show impairment in kidney function. Given the potential implications for clinical practice and public health as well, further confirmation of this finding in longitudinal studies is warranted.

## Availability of data and materials

The dataset supporting the conclusions of this article is available on request. For further information on this database, you may contact the PIs of the Confucius Hometown Aging Project (CHAP), Chengxuan Qiu (chengxuan.qiu@ki.se) or Zhongrui Yan (zhongruiy@hotmail.com).

## Abbreviations

CHAP: Confucius Hometown Aging Project
BMI: Body mass index
GDS: Geriatric Depression Scale
FPG: Fasting plasma glucose
eGFR_cys_: Cystatin C-based estimated glomerular filtration rate
MMSE: Mini-Mental State Examination
CI: Confidence interval
OR: Odds ratio
